# The integration hypothesis of human language evolution and the nature of contemporary languages

**DOI:** 10.3389/fpsyg.2014.00564

**Published:** 2014-06-09

**Authors:** Shigeru Miyagawa, Shiro Ojima, Robert C. Berwick, Kazuo Okanoya

**Affiliations:** ^1^Department of Linguistics and Philosophy, Massachusetts Institute of TechnologyCambridge, MA, USA; ^2^Center for Research and Development of Higher Education, University of TokyoTokyo, Japan; ^3^Department of Life Sciences, The University of TokyoTokyo, Japan; ^4^Department of Electrical Engineering and Computer Science and Laboratory for Information and Decision Systems, Massachusetts Institute of TechnologyCambridge, MA, USA; ^5^Okanoya Emotional Information Project, Exploratory Research for Advanced Technology, Japan Science and Technology AgencyTokyo, Japan

**Keywords:** biolinguistics, language evolution, linguistics, birdsong, agreement, movement in language

## Abstract

How human language arose is a mystery in the evolution of *Homo sapiens*. Miyagawa et al. (2013) put forward a proposal, which we will call the **Integration Hypothesis** of human language evolution, that holds that human language is composed of two components, E for *expressive*, and L for *lexical*. Each component has an antecedent in nature: E as found, for example, in birdsong, and L in, for example, the alarm calls of monkeys. E and L integrated uniquely in humans to give rise to language. A challenge to the Integration Hypothesis is that while these non-human systems are finite-state in nature, human language is known to require characterization by a non-finite state grammar. Our claim is that E and L, taken separately, are in fact finite-state; when a grammatical process crosses the boundary between E and L, it gives rise to the non-finite state character of human language. We provide empirical evidence for the Integration Hypothesis by showing that certain processes found in contemporary languages that have been characterized as non-finite state in nature can in fact be shown to be finite-state. We also speculate on how human language actually arose in evolution through the lens of the Integration Hypothesis.

## Introduction

Human language appears to have developed within the past 100,000 years (Tattersall, 2009). While it is extremely challenging to confirm any hypothesis of the actual process that led to the emergence of language, it is possible to formulate a theory that is broadly compatible with what we find in contemporary systems among mammals, birds, and humans. Miyagawa et al. (2013) put forward such a theory, which we will call the **Integration Hypothesis** of human language evolution. In this article, we will provide empirical evidence from contemporary languages for crucial components of the Integration Hypothesis. We will also speculate on how human language actually arose in evolution through the lens of the Integration Hypothesis.

We will focus on the structures found in human language and compare them to other systems such as those found in monkey alarm calls and birdsong. In recent linguistic theory, it is proposed that there is just one rule for structure building, called Merge, which takes two items and combines them into an unordered set (Chomsky, 1995). If Merge is what gives human language its unique character for building structures, it is this operation that largely distinguishes human language from other systems (Hauser et al., 2002; Berwick, 2011). This view of human language leaves open a host of questions including: (i) how did Merge appear?; (ii) why is human language characterizable by a non-finite state grammar (Chomsky, 1956) while other systems of the animal world are finite-state in nature (Berwick et al., 2011)?; and (iii) why do we find processes such as movement and agreement in human language (Chomsky, 1995; Miyagawa, 2010)? The Integration Hypothesis addresses these questions by advancing a conventional Darwinian view: two pre-adapted systems found elsewhere in the animal world were *integrated* in humans to give rise to the unique system that underlies today's languages. One system, called Type E for *expressive*, is found, for example, in birdsong (Berwick et al., 2011), which serves to mark mating availability and other “expressive” functions. The second system, Type L for *lexical*, is found in monkey calls (Seyfarth et al., 1980; Arnold and Zuberbühler, 2006) and honeybee waggle dances (Riley et al., 2005). Types E and L are the two primary forms of communication found in the animal world. Our view that human language syntax arose from pre-existing systems as found in other species is a conventional mode of evolutionary explanation, and so has been advanced by other researchers. For example, Fitch (2011) suggests that the roots of the core computational capacity of human language may be found in motor control and motor planning, while others such as Hurford (2011) allude to a gradual development from non-human primate call systems. We take no stand on these particular hypotheses regarding language's origin—directly analogizing language motor activity is not at all straightforward, as the recent exchange between Moro (2014a,b) and Pulvermüller (2014) demonstrates. Rather, we approach a different aspect of the origin of language: how a non-context free system emerged by conjoining two antecedent systems that were only finite-state. The Integration Hypothesis is advanced to explore some possibilities; it differs from other accounts like those above in that it is more linguistically detailed and broadly consistent with facts of contemporary languages. At the end, we will speculate on how the E and L systems emerged in humans.

## The integration hypothesis of human language evolution (Miyagawa et al., 2013)

Every human language sentence is composed of two layers of meaning: a *lexical* structure that contains the lexical meaning (Hale and Keyser, 1993), and an *expression* structure that is composed of function elements that give shape to the expression (Chomsky, 1995; Miyagawa, 2010). In the question, *Did John eat pizza?*, the lexical layer is composed of the words *John, eat, pizza*; these words are constant across a variety of expressions. The sentence also contains *did*, which has two functions: it marks tense, and by occurring at the head of the sentence, it also signifies a question. Tense and question are two elements that give form to the expression, making it possible to use it in conversation. The two layers of meaning are commonly represented as follows.

(1) Duality of semantics (Chomsky, 1995, 2008; Miyagawa, 2010) 
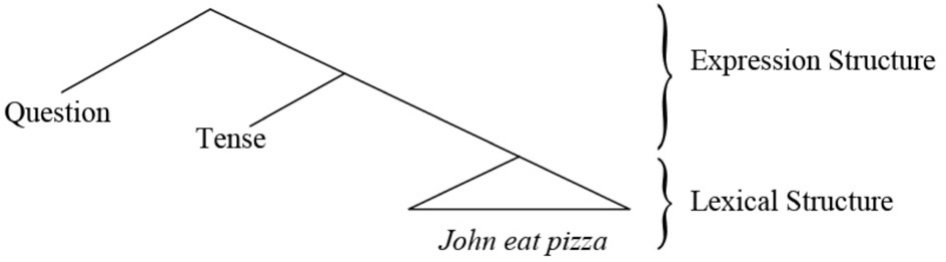

The Integration Hypothesis (Miyagawa et al., 2013) views these two layers as having antecedents in other animal species. The lexical layer is related to those systems that employ isolated uttered units that correlate with real-world references, such as the alarm calls of Vervet monkeys for pythons, eagles, and leopards (Seyfarth et al., 1980). The expression layer is similar to birdsongs; birdsongs have specific patterns, but they do not contain words, so that birdsongs have syntax without meaning (Berwick et al., 2012), thus it is of the E type. Although parallels between birdsong and human language have often been suggested (Darwin, 1871; Jespersen, 1922; Marler, 1970; Nottebohm, 1975; Doupe and Kuhl, 1999; Okanoya, 2002; Bolhuis et al., 2010; Berwick et al., 2012), we believe that the actual link is between birdsong and the expression structure portion of human language.

(2) Human language and the non-human language-like types lexical structure <—> bee dances/primate calls Type L expression structure <—> birdsong Type E

**Figure 1:**
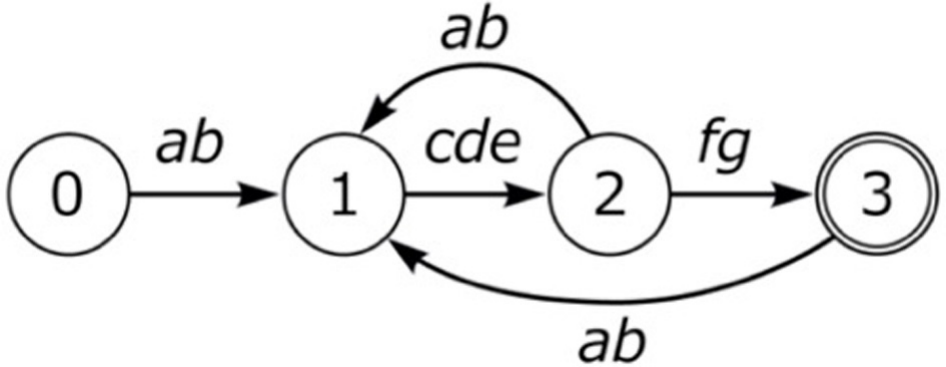
**Bengalese finch song**.

Birdsongs can be complex, as in the example of the Bengalese finch. The Bengalese finch song loops back to various positions in the song, which leads to considerable variation (Figure 1). Nevertheless, all known birdsongs can be described as a *k-reversible* finite state automaton (Berwick et al., 2011), a restricted class of automata that are efficiently learnable from examples. The L type also is a simple finite state system. The Integration Hypothesis conjectures that these two major systems in nature that underlie communication, E and L, integrated uniquely in humans to give rise to language.

Some theories of human language are not easily compatible with the views proposed here. For example, Lexical-Functional Grammar (LFG) views words and phrases as having equivalent functions. However, there are the notions of *argument structure* and *expression structure* (Bresnan, 2001, pp. 9–10) that parallel in general terms the design we are assuming. We in fact adopt the term *expression structure* from LFG. Distributed Morphology (Halle and Marantz, 1993; Marantz, 1997; Embick, 2010) denies a division between word and phrasal formation. Nevertheless, DM contains a division reminiscent of the E/L layers. “Words” are listed as category-neutral roots indicated by √, e.g., [√CONSUME]. A category specification head such as D (noun) or *v* (verb) is added to furnish category specification: [_D_ *consumption (of water)*] [_*v*_ *consume (water)*]. The “root” layer is something akin to the L system in our proposal. Once a category-specifying item is merged, that structure becomes similar to our E layer—it participates in syntactic processes of merge and labeling, movement, etc. One difference is that in DM, category-less items may combine directly, something we do not believe is possible; L items do not directly combine with each other. This is why we typically find E-L alternations^1^.

(3) E/L hierarchical structure (“D” stands for “Determiner” and is part of the E system for noun phrases) 
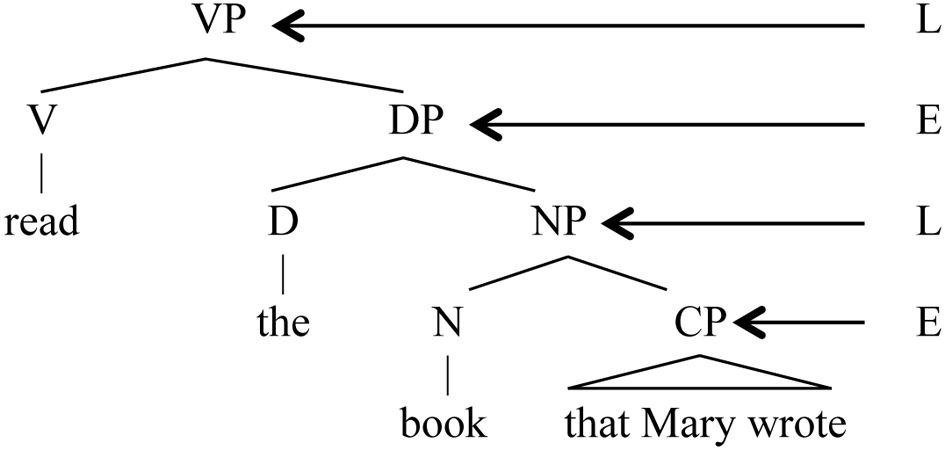

## Three challenges for the integration hypothesis from contemporary languages

We take up three challenges to the Integration Hypothesis from contemporary linguistics: two that ostensibly argue against our proposal that inside E and L we only find finite-state processes; and a third having to do with the assumption that L items cannot combine directly—any combination requires intervention from E.

The first challenge to the Integration hypothesis that E and L are finite state regards the existence of so-called discontiguous word formation. For example, Carden (1983), based on Bar-Hillel and Shamir (1960) and Langendoen (1975, 1981), argues that sequences involving the prefix *anti-* and a noun such as *missile* are non-finite state in nature (see also Boeckx, 2006; Narita et al., 2014).

(4) a. [anti-missile]
  b. [anti-[anti-missile] missile] missile

The ostensible point is that this formation can involve center embedding, which would constitute a non-finite state construction. When additional *anti* is attached to the front of the construction, one or more instances of *missile* must occur at the end (4b), giving the impression of center embedding. However, this is not the correct analysis. When *anti-* combines with a noun such as *missile*, the sequence *anti-missile* is a modifier that would modify a noun with this property, thus, [*anti-missile*]-*missile*, [*anti*-*missile*]-*defense*. Each successive expansion forms via strict adjacency, as shown by the italicized element below, without the need to posit a center embedding, non-regular grammar.

(5) a. [anti-missile]-*missile*
  b. *anti-*[[anti-missile]-missile] (modifier)
  c. [anti-[[anti-missile]-missile]]]-*missile* (or, anti-anti-missile-missile-*defense*)

The final construction also led some to claim that when *anti-* is added on the left, two instances of *missile* must occur on the right, which would be a non-regular grammar process. However, that is not the correct way to view this construction. *anti-* is attached to [[*anti-missile*]-*missile*], forming the modifier *anti*-[[*anti-missile*]*-missile*. To this the additional *missile* is added that is modified by the rest, giving appearance that two instances of *missile* were added.

The second challenge to the finite state nature of E/L is reduplication, often cited as being non-finite state (McCarthy and Prince, 1995, 1999; Urbanczyk, 2007). In reduplication a word is reduplicated in its entirety or in part.

(6) Full reduplication: C_1_V_1_C_2_V_2_C_3_ - C_1_V_1_C_2_V_2_C_3_
  Partial reduplication: C_1_V_1_ - C_1_V_1_C_2_V_2_C_3_.

Following are actual examples of full and partial reduplication (Moravcsik, 1978).

(7) a. kuuna-kuuna “husbands” (Tohono O'odham plural)
  b. tak-takki “legs” (Agta plural)

Contrary to the non-finite state approaches common in the literature, Raimy (2000) provides an analysis of reduplication that, in its most basic form, is similar to the 1 finite state automaton we saw for the song of Bengalese finch. He argues that reduplication is a process of looping back:

(8) 1 Finite State Automaton and Reduplication: 
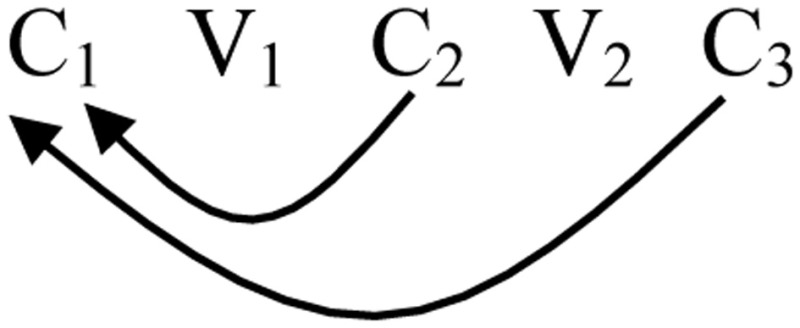

There are cases in which a reduplicant may occur to the right of the base: *erasi-rasi* “he is sick” (Siriono continuative, Key, 1965). Here the reduplicant is a copy that begins in the middle of the base and goes to the end. Right-handed reduplicants always have this property of starting in the middle of the base and copy to the end (Marantz, 1982).

(9) “Suffix” Reduplication: 
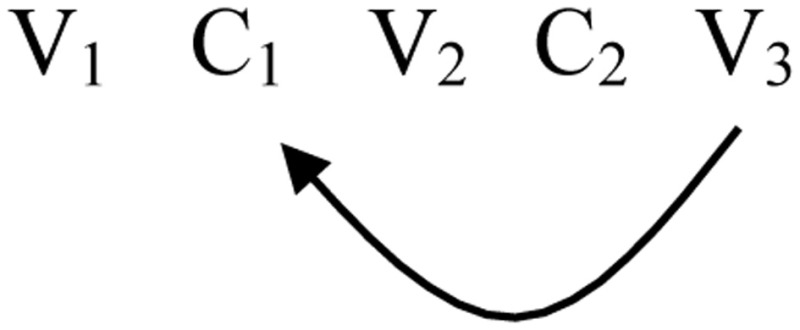

This copying process is a product of a loop back to the middle of the string.

The third challenge concerns the assumption that the members of L do not directly combine with each other. There are compound words such as *tea:cup, brain:power*, that appear to be L-L combinations. However, there is evidence that some E element does occur between the two L's. In German, when two words combine to form a compound, typically an element (/n/ or schwa) is inserted between the two words, as in *Blume-*N*-wiese* “flower meadow” (Aronoff and Fuhrhop, 2002); this “linking” element has no apparent function, so we can reasonably assume this sequence to be L-E-L. In English, we find a similar linking element in the form of /s/ in: *craft*S*man, mark*S*man, spoke*S*man* (Marchand, 1969). This /s/ has no function other than to link the two L's. These linking elements suggest that there is a slot between the two L's in compound words where we predict an E element to occur. In the case of *teacup*, where there is no overt linker, we surmise that a phonologically null element occurs in that position. As a reviewer notes, languages such as Chinese, where sentences appear to be simple noun-verb-noun sequences, the idea that there are expression items intervening between L items becomes a challenge. Sybesma (2007) argues that there are tests to detect the occurrence of tense in Chinese, hence a T head, despite the fact that it is not pronounced.

## Movement as a non-finite state process

An operation that is pervasive in human language is movement.

(10) What did you eat ___?

The question word *what* is the object of *eat*, yet it has evidently been displaced from this position of thematic interpretation after the verb to where it is actually pronounced, at the head of the sentence. This is clearly a non-finite state operation. When we look at a typical syntactic movement, it is from the L structure to the E structure: *what* begins in the L position of object, then moves to the E position of Question (e.g., Chomsky, 2001, 2008; Miyagawa, 2010).

(11) Movement 
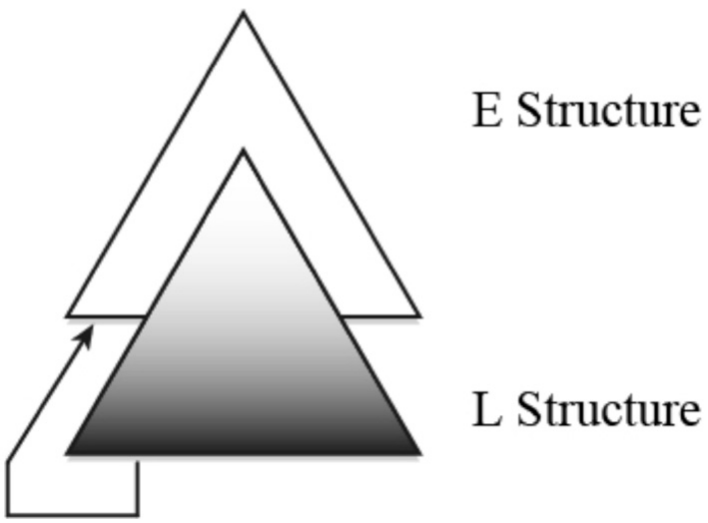

Agreement is another process that crosses E and L (Miyagawa et al., 2013). Movement and agreement are processes that, by connecting E and L, tie the two structures together. Hence, while we find finite state grammar processes inside E and L, thus reflecting their antecedents in the non-human animal world, non-finite state procedure is introduced to link the two structures. It is only in crossing from one structure to another that something other than a finite state operation is required.

Theories that do not posit movement nevertheless have operations that cross E and L. For example, Head-driven Phrase Structure Grammar (HPSG) constructs “pointers” between “what” at the head of sentences to the position after “eat,” via the propagation of information from “what” to this thematic argument point. Although there is no explicit “movement,” the effect is the same (Sag et al., 2003). Similarly, LFG reconstructs such pairings by means of information structure pairings that cross E-L boundaries, using a base context-free grammar that is composed from two finite-state systems in just the manner suggested above. To be sure, given the wide range of current syntactic theories, in other cases it is simply not possible to mimic the E-L account—an unsurprising outcome, since such theories are often incompatible with each other, as noted by Jackendoff (2010).

## Speculation on the integration of E and L

Given the evolutionary proximity between humans and other primates, the lexical structure in human language can plausibly be traced to non-human primates and their alarm calls and similar L systems. However, the same cannot be said of expression structure and birdsong. The ancestors of present-day birds and mammals split 300 million years ago (Benton, 1990), an evolutionary divide of 600 million years that suggests convergent evolution—independent evolution of E systems in birds and humans, rather than descent from a common ancestor that possessed this trait. Further, even within the *Aves* lineage, vocal learning in songbirds has been independently evolved; for example, there are closely related bird species, such as Ruby Throated hummingbird and Anna's hummingbird, where the former possesses vocal learning but the latter does not—a concrete example of convergent evolution. The other evolutionary possibility is that E systems were present in the common ancestors of humans and non-human primates, or even the rest of the mammalian lineage, in which case humans would have E in virtue of common descent, although the E system would not necessarily be expressed as part of a communication system.

Some behavioral patterns of non-human mammals can be described by finite-state grammars. Examples include the food-hoarding behavior of Syrian golden hamsters (Jones and Pinel, 1990) and the facial grooming actions of rats (Berridge et al., 1987). However, the finite-state nature of rodents' action sequences does not, in itself, make them Type-E systems. Individual action units in such sequences are relatively independent of each other, while song elements in birdsong are produced rapidly in succession, creating a sustained pattern when seen as a whole. In rodents, each action unit also has a functional meaning, while individual song elements of birds are meaningless.

The two requirements for an E system are:

(12) E System
  (i) It creates a sustained pattern;
  (ii) It holistically expresses an internal state of the singer.

E systems may be present to a limited extent in the singing behavior of non-human primates. Most non-human primates do not sing, but there is an exception: gibbons (Hylobatidae) (Marshall and Marshall, 1976; Haimoff, 1984). They sing long, complex songs. The gibbon song, as a whole, has functions such as territory advertisement, mate attraction, the strengthening of pair and family bonds (Brockelman and Srikosamatara, 1984; Raemaekers et al., 1984; Mitani, 1985; Geissmann and Orgeldinger, 2000). This is analogous to birdsong, a Type E system, which holistically expresses the singer's internal state.

In most gibbon species, male songs can be flexible in the order of notes (song elements) (Raemaekers et al., 1984; Haimoff, 1985; Mitani, 1988). For example, the male song of the Javan silvery gibbon (*Hylobates moloch*) contains 14 distinct note types, which can be assembled into a song in various orders (Geissmann et al., 2005). The transition from one note type to another appears to be probabilistic (see Figure 7 of Geissmann et al., 2005). The gibbon song, characterized by probabilistic transitions among different note types but lacking internal syntactic hierarchy, may be analogous in its grammatical structure to certain birdsong.

Hence, non-human primates, our close relatives, may have the latent potential to vocalize continuously in a finite state fashion to convey a holistic message. What prevents most of them from doing so is not entirely clear. It may be difficult for them to coordinate various articulation apparatuses rhythmically, which is required in singing and speech-like vocalizations. Non-human primates' ability to produce rhythmic orofacial movements has only recently begun to be reported. The gelada, a non-human primate, can vocalize during the action of “lip-smacking” (rapid opening and closing of the mouth and lips), which shares rhythmic features with orofacial movements involved in human speech (Ghazanfar et al., 2012; Bergman, 2013). Further searches for E-like systems should be continued in both vocal and non-vocal domains. We also need to understand the neural mechanisms underlying Type-L and Type-E systems, in evolutionary contexts. Rauschecker's work (e.g., Rauschecker, 2012) suggests that auditory regions of the brain are hierarchically organized in both humans and non-human primates, with more anterior portions of the ventral auditory stream responding to more complex auditory objects such as spoken words in humans and calls in monkeys. It might be tempting to link Type-L systems to the ventral auditory stream, but we must await future research before accepting such a view.

### Conflict of interest statement

The authors declare that the research was conducted in the absence of any commercial or financial relationships that could be construed as a potential conflict of interest.

## Footnotes

^1^As a reviewer notes, a recent approach called nanosyntax (e.g., Starke, 2009) appears to be fundamentally in conflict with the Integration Hypothesis. Nanosyntax posits that morphemes may consist of several terminal nodes, thus, syntactic in nature. We leave any attempt to compare this with our approach for future research.
